# Systematic review of Aboriginal and Torres Strait Islander peoples’ experiences and supportive care needs associated with cancer

**DOI:** 10.1186/s12889-024-18070-3

**Published:** 2024-02-20

**Authors:** John Gilroy, Mandy Henningham, Drew Meehan, Farhana Nila, Joanna McGlone, Amanda McAtamney, Kate Whittaker, Bena Brown, Megan Varlow, Tanya Buchanan

**Affiliations:** 1 Charles Perkins Centre, The University of Sydney, Sydney, NSW 2006 Australia; 2 Faculty of Medicine and Health, The University of Sydney, Sydney, NSW 2006 Australia; 3 Cancer Council Australia, Sydney, NSW 2000 Australia; 4 Inala Indigenous Health Service, Metro South Health, Inala, QLD 4077 Australia; 5 School of Health and Society, Faculty of the Arts, Social Sciences and Humanities, University of Wollongong, Wollongong, NSW 2522 Australia

**Keywords:** Systematic review, Aboriginal and Torres Strait Islander, Indigenous, Thematic analysis, Cancer, Sovereignty

## Abstract

**Background:**

Persistent disparities exist between Aboriginal and Torres Strait Islander peoples (the Indigenous peoples of Australia) and non-Indigenous Australians associated with cancer, with Aboriginal and Torres Strait Islander peoples experiencing a longer time to treatment, higher morbidity rates, and higher mortality rates. This systematic review aimed to investigate findings and recommendations in the literature about the experiences and supportive care needs of Aboriginal and Torres Strait Islander peoples with cancer in Australia.

**Methods:**

A qualitative systematic review was conducted using thematic analysis. Database searches were conducted in CINAHL, Informit, MEDLINE, ProQuest, Scopus, and Web of Science for articles published between January 2000 and December 2021. There were 91 included studies which were appraised using the Mixed Methods Appraisal Tool. The included studies reported on the experiences of cancer and supportive care needs in Aboriginal and Torres Strait Islander populations.

**Results:**

Six key themes were determined: Culture, family, and community; cancer outcomes; psychological distress; access to health care; cancer education and awareness; and lack of appropriate data. Culture was seen as a potential facilitator to achieving optimal cancer care, with included studies highlighting the need for culturally safe cancer services and the routine collection of Aboriginal and Torres Strait Islander status in healthcare settings.

**Conclusion:**

Future work should capitalize on these findings by encouraging the integration of culture in healthcare settings to increase treatment completion and provide a positive experience for Aboriginal and Torres Strait Islander peoples with cancer.

**Supplementary Information:**

The online version contains supplementary material available at 10.1186/s12889-024-18070-3.

## Introduction

Aboriginal and Torres Strait Islander (Indigenous) populations of Australia experience higher rates of both cancer morbidity and mortality than the non-Indigenous population in Australia [1]. This is consistent with similar findings of worse health outcomes for Indigenous populations from Canada, America, and New Zealand among others [2]. Improving health outcomes experienced by Aboriginal and Torres Strait Islander peoples should be a priority for both government and non-government organizations [3, 4]. However, there needs to be an awareness of the cultural values of the Aboriginal and Torres Strait Islander populations when designing interventions, services, and programs to improve cancer outcomes, and to narrow the disparity gap [4].

For Aboriginal and Torres Strait Islander peoples, health is more than a physical domain, recognizing the interplay between the physical, emotional, social, spiritual, and cultural elements that influence a person’s health [5]. Some factors that influence how Aboriginal and Torres Strait Islander peoples may experience a cancer journey include their cultural values, differences in health literacy, discrimination based on race or socioeconomic status, communication differences, and geographic isolation [6], all of which may impact on health care access and treatment. In a recent report from the Australian Institute of Health and Welfare [3], over one-third of Aboriginal and Torres Strait Islander people reported that they did not access health services when they needed them due to cultural reasons such as the lack of cultural appropriateness of the service; while one in five Aboriginal and Torres Strait Islander people reported racial discrimination by a healthcare professional in 2019–20 [3]. As a result, Aboriginal and Torres Strait Islander peoples are five times more likely than non-Indigenous people to discharge themselves from hospital earlier than recommended [3]. To combat this, there is an increasing number of services, including Aboriginal Community Controlled Health Organisations (ACCHOs), which offer culturally respectful cancer services such as bush medicine and traditional healing, alongside Western services, to support engagement and access to optimize health outcomes [7].

Aboriginal and Torres Strait Islander peoples are more likely to be exposed to risky health behaviors as a result of systemic racism and substantial socioeconomic disadvantage which will affect health decisions and behaviors [8]. These risky health behaviors include increased tobacco use, increased alcohol use, lower levels of physical activity, and poorer diet than non-Indigenous peoples [9, 10]. They are also less likely to engage in regular cancer screening programs than non-Indigenous peoples, which can lead to later diagnosis, and thereby worse cancer outcomes. In 2018–19, just 26% of Aboriginal and Torres Strait Islander women over the age of 40 presented for a mammogram, compared to 35% of non-Indigenous women. Similarly, only 40% of eligible Aboriginal and Torres Strait Islander females had participated in the national cervical screening program, compared to a participation rate of 68% in the total population [10, 11]. Only 21% of Aboriginal and Torres Strait Islander peoples who were invited to complete a bowel cancer screening test in 2016–17, had done so by June 2018, as opposed to 43.3% of non-Indigenous people [12]. Work is underway to optimize participation in these screening programs to align with the preferences of Aboriginal and Torres Strait Islander people such as widespread promotion of self-collection for cervical screening, and culturally specific communications used to promote participation in the bowel cancer screening program [13–16].

Despite the significant disparities experienced by Aboriginal and Torres Strait Islander peoples, to the best knowledge of the authors, no comprehensive review of the literature has been undertaken to understand Aboriginal and Torres Strait Islander peoples’ experiences and supportive care needs relating to cancer. A rigorous systematic review is needed to inform future health policy to improve these disparities. To address this, we aimed to conduct a thematic review of the literature on the experiences and supportive care needs of Aboriginal and Torres Strait Islander peoples with cancer in Australia. This review does not intend to focus on the diagnostic, prognostic or treatment processes associated with cancer beyond their experiential outcomes.

*NB: This article most frequently uses the term ‘Aboriginal and Torres Strait Islander peoples’ for Australia’s Indigenous peoples. However, some examined studies use the term ‘Aboriginal’ or ‘Indigenous’ which will be maintained if used by the original authors.*

## Methods

The concept for this systematic review was developed and led by A/Prof John Gilroy and Dr. Mandy Henningham, with support from the Cancer Council Australia Cancer Control Policy team. John Gilroy is a Yuin man from the NSW South Coast and is a Professor of Sociology in Indigenous Health, specializing primarily in disability studies. John is passionate about Aboriginal-owned and driven research to influence policy. Mandy Henningham is an Indigenous woman living on Dharug country in NSW. Mandy is a lecturer at the University of Sydney where they are a dedicated LGBTIQA + advocate and researcher in sexuality, sexual health, Indigenous studies, Intersex studies, youth, and mental health in the Department of Sociology and Social Policy. They bring a multidisciplinary lens to their projects which gives greater insights into the lived experiences of marginalized populations.

The Cancer Council Australia Cancer Control Policy team (DM, JM, AM, KW, MV and TB) bring a multi-disciplinary lens to this work with strong backgrounds in healthcare policy production across the cancer continuum from prevention to survivorship and end-of-life care. 

This review employs systematic reviewing methods drawn from the Joanna Briggs Institute for the literature search and is reported against the 2020 PRISMA guidelines where appropriate. We have used a qualitative thematic approach for data analysis and synthesis. The protocol for the study was published on the Cancer Council website and is available here: https://www.cancer.org.au/assets/pdf/aboriginal-cancer-care-system

### Search strategy and inclusion criteria

This systematic review included peer-reviewed scientific studies related to the experiences and supportive care needs of Aboriginal and Torres Strait Islander peoples and cancer. Six databases were used for the searches as per the research protocol: CINAHL, Informit, MEDLINE, ProQuest, Scopus, and Web of Science. Further grey literature searches were conducted through Google Scholar. Searches were carried out in January 2022. Included studies were published between January 2000 and December 2021, and were required to have been published in English. Broad search terms used included “cancer”, “neoplasms”, “Australia”, “Indigenous”, and “Aboriginal and Torres Strait Islander”, with further details of the search strategy found in Table 1.

**Table 1:** The generalized search strategy used for database searching

Line #	Searches
**1**	exp Neoplasms/ OR Neoplasm*.mp. OR Cancer*.mp. OR Malignan*.mp. OR exp Carcinoma/ OR Carcinoma*.mp. OR oncolog*.tw
**2**	Oceanic Ancestry Group/ OR Oceanic Ancestry Group*.mp OR Aborigin*.mp OR Torres Strait Islander*.mp OR indigen*.mp OR First Nation*.mp
**3**	exp Australia/ OR Australia.mp OR New South Wales.mp OR Victoria.mp OR Queensland.mp OR Northern Territory.mp OR Tasmania.mp OR Australian Capital Territory.mp OR Western Australia.mp. OR South Australia.mp OR NSW OR VIC OR NT OR SA OR WA OR QLD OR TAS OR ACT.tw

Inclusion criteria for peer-reviewed literature required the research to have involved or have detailed any Australian Aboriginal and Torres Strait Islander population and to have assessed outcomes related to a cancer diagnosis including experiences and/or supportive care needs. Studies detailing original qualitative, quantitative or mixed methods research on primary or secondary data were included. Opinion pieces, viewpoints, perspectives, invited comments, and case studies were excluded from this review. Research that was focused solely on diagnostic, prognostic or treatment processes associated with cancer was also excluded. Grey-literature was assessed against the same inclusion criteria.

### Screening and study selection

Articles were imported directly into Covidence for removal of duplicates. Title and abstract screening was conducted independently by two researchers (JG, FN) using the same software. Selected studies were full-text reviewed by three researchers together (JG, MH, FN) and then imported into Endnote for reference management. All conflicts were resolved by the two lead researchers (JG, MH) through discussion.

### Data synthesis

Data charting was completed for all included studies according to the following criteria: authors, year of publication, title, peer review status, aims, methods, data source, participants, and key findings.

Further to this data charting, a qualitative thematic analysis of the text of the included studies was conducted between February and June 2022, using the reflexive thematic analysis process developed by Braun and Clarke [17]. Coding was completed by three researchers (JG, MH, FN) using NVIVO, and codes and key themes were defined inductively.

### Data quality appraisal

The appraisal was conducted by three researchers (JG, MH, FN) using the Mixed Method Appraisal Tool (MMAT) (version 2018) due to its ability to appraise multiple methodologies [18]. A further check of the appraisal was conducted by two researchers to ensure validity (DM, JM).

## Results

**Fig. 1:**
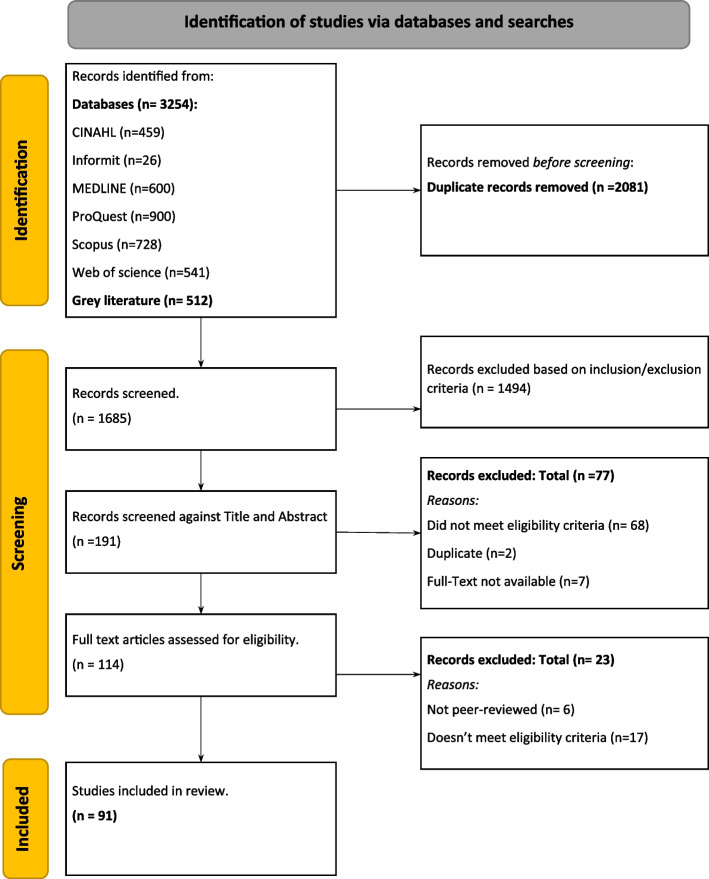
PRISMA flowchart of the screening process

A total of 3766 records were retrieved from databases, with 2081 duplicates removed before screening. The remaining 1685 articles were screened by title and abstract based on the inclusion/exclusion criteria, with 1494 articles not meeting inclusion criteria being excluded. After the full-text screening, authors agreed on the final ninety-one articles which have been included in this systematic review. The PRISMA flowchart of this process is below (Fig. 1) [19].

### Characteristics of included studies

**Fig. 2:**
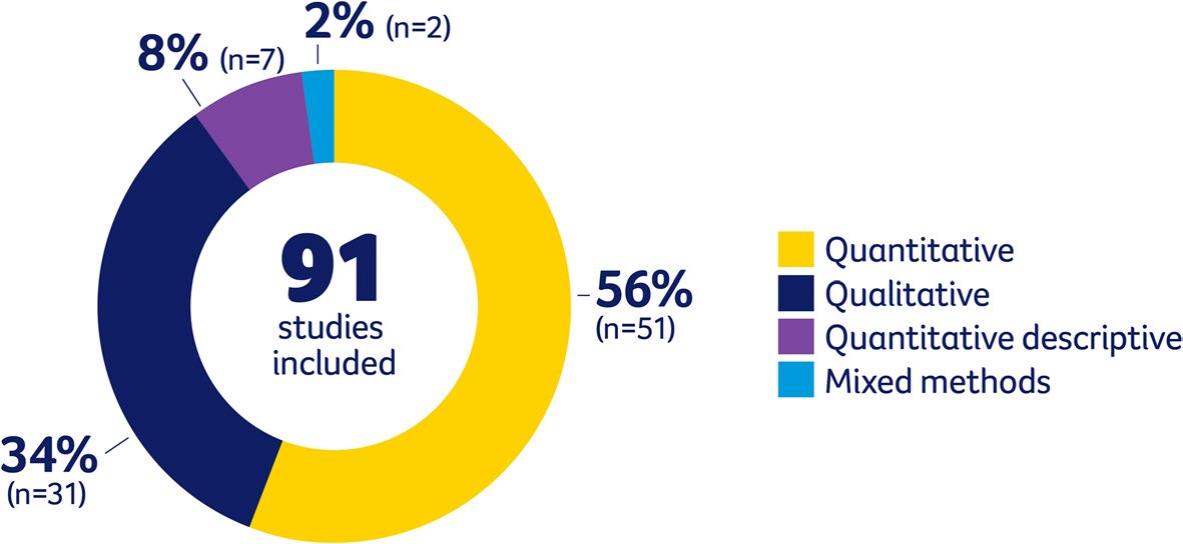
Study designs of the included 91 studies

Of the 91 studies included in this review, 56% (*n* = 51) were quantitative studies, 34% (*n* = 31) were qualitative, 8% (*n* = 7) were quantitative descriptive, and 2% (*n* = 2) were mixed methods studies (See Fig. 2). Most of the qualitative studies aimed to explore the perceptions, beliefs, and cancer care experiences of Aboriginal and Torres Strait Islander peoples living with cancer and possible barriers to accessing care [20–38], understanding the perspectives of health care providers and caregivers who provide care to Aboriginal and Torres Strait Islander peoples [39–43], and to describe the role of Aboriginal and Torres Strait Islander health care workers in providing culturally appropriate health care services [44–46]. Of the quantitative studies, 5% (*n* = 5) assessed outcomes in Aboriginal and Torres Strait Islander children specifically [47–51]. It was common among the included quantitative studies to assess survival outcomes and disparities. Over half (*n* = 4) of the quantitative descriptive design studies included assessed healthcare utilization [52–55].

### Quality appraisal

Using the MMAT tool to assess the methodological quality of the included papers, almost all quantitative, quantitative descriptive, and qualitative papers (*n* = 87) met all the criteria. Two mixed methods studies did not adequately address all methodological quality criteria due to divergences and variations in the reporting of their methods. The detailed mixed-method appraisal of the included studies can be found in additional material (See Additional file 2).

### Thematic analysis

The key themes identified in the thematic analysis were: Culture, family, and community; cancer outcomes; psychological distress; access to health care; cancer education and awareness; and lack of appropriate data. Table 2 provides quotes for each theme.

**Table 2:** Generating themes from supporting quotes

Themes	Codes	Quotes
**Culture, family, and community**	Culturally safe care services	*“… the rooms are often too small for when family visits. They [Yamatji] have large families and prefer to sit outside in the garden during visiting hours. We only have two single rooms and having another person in the room doesn’t allow for much privacy, that’s why they also prefer the garden.”* (Participant 1) [20].
	Communication barrier	*“Sometimes we have to use family members which isn’t ideal because, again, it’s all the understanding and relaying—you’ve no idea of what’s actually been said to the patients in terms of emphasis.”* – CCP, 01 [23]. *“Sometimes people would come down here and they would die in hospital and it would be very traumatic for everybody, and the staff are watching these people cry and be really upset and not be able to talk to them in the same language, so that’s very distressing for everybody.”* (P14, female remote nurse) [46].
	Aboriginal healthcare worker and liaison officer	*“[The ILO] helps me to understand what the doctors are telling me. I need her to help because I don’t have escort with me.” [Patient interview] *[56].
	Involving family and community	*“Yeah, well, that’s our culture. Like, when someone is close to passing away the whole extended family will come. That’s been like that for years and years. You can’t change that, you know”* (Urban, female patient) [37].
**Cancer outcomes**	Late or advanced stage at diagnosis	*“We know the mainstream doesn’t really work for our sick people. They are going to hospital because it’s a last resort …. They are sick. That’s why sometimes it’s too late for a lot of Aboriginal people because there is not culturally appropriate cancer clinics or surgeries.”* (Urban Aboriginal HSP) [33].
	Effects of comorbidity	*“We have shown that increased comorbidity is associated with reduced five-year cause-specific and all-cause survival for Australian women diagnosed with cervical cancer.” *[57].
	Delay in treatment commencement or completion	*“Indigenous Australians are modestly more likely than other patients to experience delay between being designated ready for care and commencing the episode of care.”* [58].
	Coping with cancer and poor QOL of cancer survivors	*“And then it’s just funny how things turn out you know that after all, you know you get one (breast) taken off... I’ve accepted that I’ve had a mastectomy and like I walk around home with no shirt on you know, I’ve accepted it”* (#2693) [34].
	Culturally sensitive Allied health services	*“They said they’d put dietitians and nutritionists to talk to me and psychiatrists and psychologists, but talking to me isn’t working, it isn’t doing anything. It’s frustrating me, and it makes me angry.”* PT, 16 [23].
**Psychological distress**	Anxiety	*“I think it’s probably just traumatic, … it’s traumatic for metro people to come to the hospitals and have treatment—but especially rural communities, the whole Perth [city] situation is traumatic”* (rural Aboriginal family member) [33].
	Fear	*“There’s a lack of trust and a fearing of the hospital, so people present late, when they present late, they then die, and the cycle goes on and on.”* (P18, female nurse) [46].
	Shame	*"…it was almost like you deserved it or there was definitely this sense of shame. It was whispered. If someone died of a heart attack you would say that, but… all this cancer stuff was a whispered sort of stuff." *[32].
	Financial stress	*“[Indigenous] people are coming away from their communities – they have money issues, family issues, job issues, you name it. Let alone dealing with their cancer.”* [Care provider, CP022] [56].
**Access to healthcare**	Geographic diversity	*“If I had to go to Brisbane for lung treatment, I would not go. Neither would others, especially Indigenous. Getting down to Brisbane is very hard.”* (Quote from a community member) [59].
	Transport to health care center	*“I’ve got a lady who comes down and every time she asks me to get her an earlier (appointment)… she comes from x [4 h away from Brisbane], so she leaves so early in the morning and she doesn’t get home until late at night”.* Hospital ALO 7 [41].
	Flexible cancer care and telehealth services	*“ care providers in the current study recognized that flexibility in the duration, timing, location, and number of attendees of the consultations, was imperative to ensuring culturally relevant and appropriate care for Indigenous cancer patients.”* [56].
	Difficulty in access, continuation, and coordination of health care services	*“I have a lot of women calling me because they don’t know what services they can claim. They don’t know that they can get a bed at home or have a nurse come and change their dressing. Most of the time I tell them who to ring up and help them with the paperwork.”* (Participant 5) [20].
**Cancer education and awareness**	Misconception and myths	*“[t]o my experience I – like I learn a lot of people, Aboriginal people, like believe in Aboriginal culture so … Yeah, like a curse, and I think beliefs come from old days. Cultural side … strong belief in spirits and I guess black magic or whatever you want to call it..” *[24].
	Aboriginal understanding of cancer	*“I think still a small proportion that believes that cancer can be like a punishment of… the things that you have done in the past and that’s why you have to suffer past things in life. Like a karma type thing.”* (R2) [21].
	Limited cancer knowledge	*“... with the remote Aboriginals in the community... they go home and take this medication and they haven’t got a clue what they are taking or what it is for or anything. If you don’t understand why you have to do something, sometimes you don’t do it.”* — Rural female family member [32].
**Lack of appropriate data**	Lack of Aboriginal and Torres Strait Islander status recording	*“It was proposed that perhaps Aboriginal patients are attending but that these attendances are not reflected in the statistics. This may be because patients are not identifying as Aboriginal or because their status is not being recorded due to inadequate processes.” *[22].
	Inadequate Aboriginal and Torres Strait Islander specific cancer data	*“The NT Indigenous incidence rates reported here under-estimate actual cancer incidence by approximately 15–20%, because of a small degree of under-ascertainment of cases and misclassification of Indigenous status in the NTCR.” *[60].

#### Theme one: culture, family, and community

The thematic analysis identified several concerns influencing the experiences of cancer for Aboriginal and Torres Strait Islander peoples relating to cultural insensitivity, including lack of culturally appropriate care services, language barriers, poor understanding of Aboriginal and Torres Strait Islander peoples’ perspectives about cancer and the importance of family and community involvement [22, 27, 32, 40, 46, 61–64]. The majority of the included studies reported that care providers lacked an understanding of Aboriginal and Torres Strait Islander culture and how this shaped treatment decisions [42, 46, 65]. Included studies highlighted value differences between Aboriginal and Torres Strait Islander cultures and Western cultures [26, 27, 56]. Prioritizing family commitments and living in a community, were frequently stated as more important than health concerns for Aboriginal and Torres Strait Islander peoples [26, 27, 56].

#### Theme two: cancer outcomes

The second theme identified cancer outcomes. Subthemes included advanced stage at diagnosis, presence of co-morbidities, longer time between diagnosis, and treatment commencement. Aboriginal and Torres Strait Islander peoples with cancer were more likely to present with distant metastasis or stage IV cancer which contributed to a lower survival rate [66–72]. Included studies mentioned that the delayed presentation of Aboriginal and Torres Strait Islander peoples with cancer was attributed to several causes, including lack of outreach services, communication barriers, complex health care system, limited health literacy, and fear and mistrust associated with cancer and its treatment [22, 56, 66, 71]. When analysing the burden of co-morbidities on cancer outcomes, a high proportion of included studies found that higher levels of co-morbidity were interweaved with poor treatment compliance and survival disadvantage; noting that poor treatment compliance does not imply a strengths-based approach, but is rather the dominant terminology used in the reviewed articles [68, 73–76]. Studies have found that the effect of co-morbidity varies with type and stage of cancer and more research is needed to explain the survival disparities of particular cancer types [57].

#### Theme three: psychological distress

Multiple stressors including financial worries, anxiety, fear, shame, and family role, in conjunction with a mistrust of non-traditional treatments, play important roles in shaping the perceptions of Aboriginal and Torres Strait Islander people with cancer. Included studies identified that worry about out-of-pocket costs related to accommodation and transport to and from hospitals were major unmet supportive care needs among Aboriginal and Torres Strait Islander people with cancer [29, 35, 43, 77–79]. Emotional strain and anxiety related to accessibility and affordability of culturally appropriate care services, pre-existing health issues, invasive treatment and follow-up, and end-of-life care were associated with late diagnosis and poor prognosis [23, 36, 80–82]. Publications highlighted the need for psychological and emotional support to reduce cancer disparities among Aboriginal and Torres Strait Islander peoples, such as survival, mortality and incidence [26, 37, 45, 81, 83]. Included studies described how psychosocial factors like fear and shame can impact the length of treatment, as well as the type of treatment that is accessed by Aboriginal and Torres Strait Islander peoples with cancer, therefore impacting their survival [21, 24, 25, 27, 31, 46].

#### Theme four: access to healthcare

Distance to travel to cancer services, worry about transport to access healthcare services, and the necessity for more flexible care services were identified in two-thirds of the included studies. Other concerns raised were difficulty in access, coordination and continuation of cancer care, and a lack of Aboriginal health care workers [46, 61, 64]. Studies found that survival disparities of Aboriginal and Torres Strait Islander peoples with cancer increased proportionately with socio-economic disadvantage and geographic remoteness [48, 51, 84–87].

Researchers identified flexible supportive care services such as after-hour services, drop-in clinics, and telehealth services as enablers to improved accessibility and continuity of cancer treatment [20, 35, 43, 56]. Included studies mentioned the need for clear navigation of scheduling, booking, and follow-up information about appropriate treatment and allied health services [37, 41, 76, 81, 86]. Developing a trusting relationship with physicians and health care workers, and provisions to support seeing the same health practitioner during follow-up visits were identified as vital enablers to facilitate the continuity of care [30, 42, 47, 88, 89].

#### Theme five: cancer education and awareness

Cancer awareness, information and knowledge impacted the time taken to seek cancer treatment in included publications [21, 23, 33, 53, 54, 90, 91]. The median time taken to seek cancer treatment was found to be significantly higher in Aboriginal and Torres Strait Islander peoples with cancer than non-Indigenous people with cancer [89]. Quotes presented in Table 2 demonstrate how different cultural beliefs and concepts like cancer is ‘contagious’, it’s a ‘payback’, means ‘bad luck’, caused by ‘black magic’, make Aboriginal and Torres Strait Islander peoples with cancer reluctant to talk about their symptoms and to ignore the warning signs [21, 22, 24, 27, 31, 52].

#### Theme six: lack of appropriate data

Included studies mentioned poor recording of Aboriginal and Torres Strait Islander status when accessing cancer treatment, therefore limiting the availability of accurate data [55, 76, 92–95]. One study found that 23% of Aboriginal and Torres Strait Islander peoples with cancer had medical records that lacked information about the stage of lung cancer at diagnosis indicating a gap in the information collected [92]. The concern of the inadequate health-related quality of life (HRQoL) data for Aboriginal and Torres Strait Islander peoples with cancer has also been identified, which is considered a benchmark tool to analyse present cancer care and the patient experience [96].

## Discussion

This review aimed to investigate and synthesize the findings and recommendations about the experiences, outcomes, and supportive care needs of Aboriginal and Torres Strait Islander peoples with cancer. The studies included in this review identified many disparities between Aboriginal and Torres Strait Islander peoples and non-Indigenous people with cancer including later diagnoses, delays in treatment, and higher rates of co-morbidity [57, 70, 88]. The need for culturally safe healthcare sites and services was highlighted in the included studies, as was the need for better-designed targeted interventions and provisions for routine data collection [41].

There is a lack of appropriate data collected about Aboriginal and Torres Strait Islander peoples with cancer. The recording of Aboriginal and Torres Strait Islander status in health data repositories is commonly inadequate, with linked data sets still routinely misclassifying a significant proportion of Aboriginal and Torres Strait Islander peoples [97]. The current research is limited by inadequate and missing routinely collected data in national administrative and health datasets, and research datasets such as surveys reporting on health-related quality of life outcomes for Aboriginal and Torres Strait Islander peoples [97]. National best practice standards exist to guide the routine collection of Indigenous status in health data sets, and other organisation level research has identified strategies to increase Aboriginal and Torres Strait Islander identification when collecting data [98, 99]. Providing opportunities for people to indicate their Aboriginal and/or Torres Strait Islander status should be a priority of all healthcare organisations, healthcare professionals and researchers [97]. Having accurate records of Aboriginal and Torres Strait Islander peoples in Australia accessing healthcare, can help to ensure greater understanding of the healthcare issues and needs faced by Aboriginal and Torres Strait Islander peoples, and can ensure the provision of culturally appropriate health services tailored to these needs.

Strong spiritual and cultural beliefs can also act as a barrier to optimal cancer care as there are longstanding attitudes that cancer is taboo and something to be ashamed of [24, 31]. The impact that these cultural beliefs have on the way that Aboriginal and Torres Strait Islander peoples may perceive their diagnosis can also reduce the effectiveness of educational campaigns about significant risk factors such as tobacco and alcohol use [100]. These strong cultural beliefs may prevent people from participating in routine cancer screening out of avoidance, shame, and a lack of education about the benefits [33, 82, 101]. These attitudes, in turn, may prevent those diagnosed with cancer from accessing the recommended care and treatment [31, 82].

Similar to previous studies, the findings of this review found that a key enabler to Aboriginal and Torres Strait Islander participation in treatment services is provision of care that respects Aboriginal understanding of cancer and acknowledges the priority of family, community, and staying connected to country [27, 62, 64, 102]. Creating culturally aware spaces for Aboriginal and Torres Strait Islander people undergoing cancer treatment was noted by many of the reviewed studies as being important to support Aboriginal and Torres Strait Islander peoples accessing timely and appropriate care [20]. Several studies also highlighted the role of Aboriginal liaison officers to ensure cultural appropriateness, treatment continuity, and co-ordination [42, 56, 65]. Likewise, included studies investigated improving communication between the health care professionals and Aboriginal and Torres Strait Islander people affected by cancer, to strengthen treatment participation and adherence [32, 46, 53]. Education about cancer was identified as an important factor in supporting the provision of optimal care to Aboriginal and Torres Strait Islander peoples in studies included in this review [31, 44, 56]. Educational tools which employ culturally specific information and contain the voices of Aboriginal and Torres Strait Islander peoples prove to be more effective than more generalized advice in supporting healthy behaviour changes [31, 61]. Researchers suggested that it is imperative to incorporate culturally relevant information and approaches into the design of cancer awareness programs, specific cancer information sheets and brochures for Aboriginal and Torres Strait Islander communities, to both facilitate an accurate understanding of cancer information and to support cultural values [21, 33, 53, 102]. The provision of health education to Aboriginal and Torres Strait Islander communities is another critical component in increasing the understanding of cancer by improving health literacy, which can help to dispel the stigma and shame that arises from a misunderstanding of cancer. As such, healthcare services, and other community organisations must be equipped and supported to provide information, education and culturally appropriate spaces for Aboriginal and Torres Strait Islander peoples who have received a cancer diagnosis.

In the reciprocal, education for healthcare practitioners on the cultural values and beliefs held by Aboriginal and Torres Strait Islander peoples was also found to be vital to ensuring culturally safe and appropriate cancer care. Previous reviews have found that cultural competency training for healthcare professionals can improve the health behaviours of their culturally diverse patients [103]. Cultural competency training, while not the only solution needed, is an important step in helping non-Indigenous healthcare professionals understand the intricacies of the Aboriginal and Torres Strait Islander peoples’ culture and how it can either help or hinder cancer care provision [30, 104]. It should be acknowledged that the cultural competency of healthcare professionals is reliant on societal level actors or “owners of the system” as they determine funding and the importance that is placed on delivering the best possible care. The onus is on governments, teaching facilities like universities and on upper management of healthcare services to ensure that healthcare professionals are confident and able to provide culturally safe care [105, 106].

This review has found culture as both a barrier and enabler for optimal cancer care for Aboriginal and Torres Strait Islander peoples and highlights a need for more culturally safe services and targeted education for Aboriginal and Torres Strait Islander peoples. Policy must reflect this need, with an increased focus on access for this priority population. The provision of culturally safe services should be accessible for Aboriginal and Torres Strait Islander peoples, regardless of their geographic location. To ensure that culturally appropriate care can be accessed for Aboriginal and Torres Strait Islander peoples across Australia, there is a need to increase the availability of health services in rural and remote areas of Australia.

The findings of this review, while specific for the Australian Aboriginal and Torres Strait Islander peoples’ experiences in cancer care, could be extrapolated to other Indigenous groups in countries with similar publicly funded healthcare systems. Systematic reviews of cancer care elements in Canada’s Indigenous populations have been published, finding that the cultural safety of services, meaning the ability of services to recognise and respect the cultural needs and identities of consumers, is imperative to uptake and utilization [107–109].

### Limitations

The MMAT tool, although tested for its veracity, is limited in scope when compared to other appraisal tools. The MMAT tool does not consider how the research was conducted with participants, therefore an additional tool which applies a cultural lens to the appraisal of research conducted about Aboriginal and Torres Strait Islander peoples was considered for this project.

Another limitation of this study is that the searches were conducted in January 2022, and due to delays in its synthesis, there may be more recent articles published after this search date. The research archive is continually evolving and so this systematic review provides an evidence base for which future reviews may expand upon. 

Further, this review explicitly looked at studies of Aboriginal and Torres Strait Islander peoples with a previous diagnosis of cancer, meaning that other vital aspects of Aboriginal and Torres Strait Islander cancer research, for example cancer screening and preventative behaviours, were beyond the scope of this review and warrant further investigation. The large number of included studies shows that the research investment is producing a sound research archive on Aboriginal and Torres Strait Islander people with cancer. This investment, by both government and non-government organisations, must continue its momentum.

## Conclusions

This review described the multifaceted, far-reaching impacts that cancer has on the experience of Aboriginal and Torres Strait Islander peoples and demonstrated that Aboriginal and Torres Strait Islander peoples face many barriers to receiving optimal cancer care including difficulty in accessing culturally appropriate health services. Culture is both a barrier and facilitator to cancer care. By improving the cultural safety of health services, and by developing new programs and services to address the specific needs of Aboriginal and Torres Strait Islander peoples, it is expected that the disparity in cancer outcomes between Aboriginal and Torres Strait Islander peoples and non-Indigenous people can be significantly reduced.

### Supplementary Information

**Additional file 1: Table S1.** Bibliographic table of included studies.

**Additional file 2: Table S2.** Critical appraisal of included studies using the Mixed Methods Appraisal Tool (MMAT).

**Additional file 3: Table S3.** PRISMA 2020 guidelines for systematic reviews checklist.

## Data Availability

All data generated or analysed during this study are included in this published article [and its additional information files].

## Abbreviations

HRQoL: Health-related quality of life
MMAT: Mixed method appraisal tool
ACCHO: Aboriginal Community-Controlled Health Organization
