# Supplementary material for: Systematic review of Aboriginal and Torres Strait Islander peoples’ experiences and supportive care needs associated with cancer
Source: BMC Public Health. 2024 Feb 20;24:523. doi: 10.1186/s12889-024-18070-3 (PMC10877816; doi:10.1186/s12889-024-18070-3)
Supplement: Supplementary file 2 — Additional file 2: Table S2. Critical appraisal of included studies using the Mixed Methods Appraisal Tool (MMAT). [file 12889_2024_18070_MOESM2_ESM.docx]

**Table S2.** Critical appraisal of included studies using the Mixed Methods Appraisal Tool (MMAT).

| Citation | Title | MMAT Screening Questions | | | | | | |
| --- | --- | --- | --- | --- | --- | --- | --- | --- |
| Quantitative |  |  |  |  |  |  |  |  |
|  |  | Are there clear research questions or aims? | Do the collected data allow to address the research questions? | Are the participants representative of the target population? | Are measurements appropriate regarding both the outcome and intervention (or exposure)? | Are there complete outcome data? | Are the confounders accounted for in the design and analysis? | During the study period, is the intervention administered (or exposure occurred) as intended? |
| (Tan et al. 2016) | A prospective comparison of times to presentation and treatment of regional and remote head and neck patients in North Queensland, Australia | Yes | Yes | Yes | Yes | Yes | Yes | Yes |
| (Moore et al. 2011) | A study of head and neck cancer treatment and survival among indigenous and non-indigenous people in Queensland, Australia, 1998 to 2004 | Yes | Yes | Yes | Yes | Yes | Yes | Yes |
| (Ho-Huynh et al. 2022) | Achieving High Breast Cancer Survival for Women in Rural and Remote Areas | Yes | Yes | Yes | Yes | Yes | Yes | Yes |
| (Tervonen et al. 2017) | After accounting for competing causes of death and more advanced stage, do Aboriginal and Torres Strait Islander peoples with cancer still have worse survival? A population-based cohort study in New South Wales | Yes | Yes | Yes | Yes | Yes | Yes | Yes |
| (Gibberd et al. 2015) | Are Aboriginal people more likely to be diagnosed with more advanced cancer? | Yes | Yes | Yes | Yes | Yes | Yes | Yes |
| (Jessop et al. 2021) | Are outcomes for childhood leukaemia in Australia influenced by geographical remoteness and Indigenous race? | Yes | Yes | Yes | Yes | Yes | Yes | Yes |
| (Martin et al. 2009) | Association of diabetes with survival among cohorts of Indigenous and non-Indigenous Australians with cancer | Yes | Yes | Yes | Yes | Yes | Yes | Yes |
| (Moore et al. 2016) | Breast cancer diagnosis, patterns of care and burden of disease in Queensland, Australia (1998–2004): does being Indigenous make a difference? | Yes | Yes | Yes | Yes | Yes | Yes | Yes |
| (Roder et al. 2012) | Breast Screening and Breast Cancer Survival in Aboriginal and Torres Strait Islander Women of Australia | Yes | Yes | Yes | Yes | Yes | Yes | Yes |
| (Valery et al. 2006) | Cancer diagnosis, treatment, and survival in Indigenous and non-Indigenous Australians: a matched cohort study | Yes | Yes | Yes | Yes | Yes | Yes | Yes |
| (Rotte et al. 2013) | Cancer in Australian Aboriginal children: Room for improvement | Yes | Yes | Yes | Yes | Yes | Yes | Yes |
| (Valery et al. 2013a) | Cancer Incidence and Mortality in Indigenous Australian Children, 1997–2008 | Yes | Yes | Yes | Yes | Yes | Yes | Yes |
| (Condon, Armstrong, et al. 2005) | Cancer incidence and survival for Indigenou Australians in the Northern Territory | Yes | Yes | Yes | Yes | Yes | Yes | Yes |
| (Valery et al. 2013b) | Cancer survival in Indigenous and non-Indigenous Australian children: what is the difference? | Yes | Yes | Yes | Yes | Yes | Yes | Yes |
| (Banham et al. 2019) | Cancer treatment and the risk of cancer death among Aboriginal and non-Aboriginal South Australians: analysis of a matched cohort study | Yes | Yes | Yes | Yes | Yes | Yes | Yes |
| (Youlden et al. 2022) | Changes in cancer incidence and survival among Aboriginal and Torres Strait Islander children in Australia, 1997–2016 | Yes | Yes | Yes | Yes | Yes | Yes | Yes |
| (Banham, Roder, and Brown 2018) | Comorbidities contribute to the risk of cancer death among Aboriginal and non-Aboriginal South Australians: Analysis of a matched cohort study | Yes | Yes | Yes | Yes | Yes | Not sure | Yes |
| (Diaz et al. 2018) | Comorbidity and cervical cancer survival of Indigenous and non-Indigenous Australian women: A semi-national registry-based cohort study (2003-2012) | Yes | Yes | Yes | Yes | Yes | Yes | Yes |
| (Tomita et al. 2016) | Comparable survival outcome of metastatic colorectal cancer in Indigenous and non-Indigenous patients: Retrospective analysis of the South Australian metastatic colorectal cancer registry | Yes | Yes | Yes | Yes | Yes | Yes | Yes |
| (Cottrell et al. 2007) | Comparing Cancer Profiles and Survival of Aboriginal and non-Aboriginal Patients in South Australia: Where are the Opportunities for Improving Aboriginal Health? | Yes | Yes | Yes | Yes | Yes | No | Yes |
| (Weir et al. 2016) | Comparing colorectal cancer treatment and survival for Aboriginal and non-Aboriginal people in New South Wales | Yes | Yes | Yes | Yes | Yes | No | Yes |
| (Slape et al. 2019) | Cutaneous malignancies in Indigenous Peoples of urban Sydney | Yes | Yes | Yes | Yes | Yes | No | Yes |
| (Woods et al. 2018) | Delay in commencement of palliative care service episodes provided to Indigenous and non-Indigenous patients: cross-sectional analysis of an Australian multi-jurisdictional dataset | Yes | Yes | Yes | Yes | Yes | Yes | Yes |
| (Pule et al. 2018) | Developing a comorbidity index for comparing cancer outcomes in Aboriginal and non-Aboriginal Australians | Yes | Yes | Yes | Yes | Yes | Yes | Yes |
| (Tervonen et al. 2016) | Differences in impact of Aboriginal and Torres Strait Islander status on cancer stage and survival by level of socio-economic disadvantage and remoteness of residence—A population-based cohort study in Australia | Yes | Yes | Yes | Yes | Yes | Yes | Yes |
| (Bernardes, Langbecker, et al. 2019) | Does social support reduce distress and worry among Aboriginal and Torres Strait Islander people with cancer? | Yes | Yes | Yes | Yes | Yes | Yes | Yes |
| (Luke et al. 2010) | Exploring contrary trends in bladder cancer incidence, mortality and survival: implications for research and cancer control | Yes | Yes | Yes | Yes | Yes | Yes | Yes |
| (Diaz et al. 2015) | Factors Associated With Cancer-Specific and Overall Survival Among Indigenous and Non-Indigenous Gynaecologic Cancer Patients in Queensland, Australia | Yes | Yes | Yes | Yes | Yes | Yes | Yes |
| (Parker et al. 2014) | Hepatocellular carcinoma in Australia’s Northern Territory: high incidence and poor outcome | Yes | Yes | Yes | Yes | Yes | Yes | Yes |
| (Supramaniam et al. 2014) | Increasing rates of surgical treatment and preventing comorbidities may increase breast cancer survival for Aboriginal women | Yes | Yes | Yes | Yes | Yes | Yes | Yes |
| (Gibberd et al. 2016) | Lung cancer treatment and mortality for Aboriginal people in New South Wales, Australia: results from a population-based record linkage study and medical record audit | Yes | Yes | Yes | Yes | Yes | Yes | Yes |
| (Fitzadam et al. 2021) | Lung, breast and bowel cancer treatment for Aboriginal people in New South Wales: a population-based cohort study | Yes | Yes | Yes | Yes | Yes | Yes | Yes |
| (Carruthers et al. 2019) | Measuring (and narrowing) the gap: The experience with attendance of Indigenous cancer patients for Radiation Therapy in the Northern Territory | Yes | Yes | Not Sure | Yes | Yes | Yes | Yes |
| (Frydrych et al. 2014) | Oral Cavity Squamous Cell Carcinoma - Characteristics and Survival in Aboriginal and Non-Aboriginal Western Australians | Yes | Yes | Yes | Yes | Yes | Yes | Yes |
| (Laurvick et al. 2003) | Ovarian cancer in Western Australia (1982-98): incidence, mortality and survival | Yes | Yes | Yes | Yes | Yes | Yes | Yes |
| (Rodger et al. 2015) | Prostate cancer mortality outcomes and patterns of primary treatment for Aboriginal men in New South Wales, Australia | Yes | Yes | Yes | Yes | Yes | Yes | Yes |
| (Elder-Robinson et al. 2022) | Quality of Life in the First Year of Cancer Diagnosis among Aboriginal and Non-Aboriginal People Living in Regional and Remote Areas of Australia | Yes | Yes | Yes | Yes | Yes | Yes | Yes |
| (Baade et al. 2016) | Quantifying the changes in survival inequality for Indigenous people diagnosed with cancer in Queensland, Australia | Yes | Yes | Yes | Yes | Yes | Yes | Yes |
| (Le et al. 2013) | Radiation treatment compliance in the Indigenous population: The pilot Northern Territory experience and future directions | Yes | Yes | Yes | Yes | Yes | Yes | Yes |
| (Shaw and Elston 2003) | Retrospective, 5-year surgical audit comparing breast cancer in indigenous and non-indigenous women in Far North Queensland | Yes | Yes | Yes | Yes | Yes | No | Yes |
| (Condon, Barnes, et al. 2005) | Stage at diagnosis and cancer survival for Indigenous Australians in the Northern Territory | Yes | Yes | Yes | Yes | Yes | Yes | Yes |
| (Diaz et al. 2016) | Supportive care needs among Indigenous cancer patients in Queensland, Australia: less comorbidity is associated with greater practical and cultural unmet need | Yes | Yes | Yes | Yes | Yes | Yes | Yes |
| (Yang and Baade 2021) | Survival disparities among recently diagnosed Aboriginal and Torres Strait Islander cancer patients in Australia remain | Yes | Yes | Yes | Yes | Yes | Yes | Yes |
| (Moore et al. 2014) | Survival disparities in Australia: an analysis of patterns of care and comorbidities among indigenous and non-indigenous cancer patients | Yes | Yes | Yes | Yes | Yes | Yes | Yes |
| (Coory et al. 2008) | Survival of Indigenous and non-Indigenous Queenslanders after a diagnosis of lung cancer: a matched cohort study | Yes | Yes | Yes | Yes | Yes | Yes | Yes |
| (Whop et al. 2017) | Time to clinical investigation for Indigenous and non-Indigenous Queensland women after a high grade abnormal Pap smear, 2000-2009 | Yes | Yes | Yes | Yes | Yes | Yes | Yes |
| (Basnayake et al. 2021) | Treatment and outcomes for indigenous and non-indigenous lung cancer patients in the Top End of the Northern Territory | Yes | Yes | Yes | Yes | Yes | Yes | Yes |
| (Hall et al. 2004) | Treatment patterns for cancer in Western Australia: does being Indigenous make a difference? | Yes | Yes | Yes | Yes | Yes | Yes | Yes |
| (Bernardes, Diaz, et al. 2019) | Unmet supportive care needs among Indigenous cancer patients across Australia | Yes | Yes | Yes | Yes | Yes | Yes | Yes |
| (Valery et al. 2017) | Unmet supportive care needs of Australian Aboriginal and Torres Strait Islanders with cancer: a prospective, longitudinal study | Yes | Yes | Yes | Yes | Yes | Yes | Yes |
| (Garvey et al. 2016) | Health-related quality of life among Indigenous Australians diagnosed with cancer | Yes | Yes | Yes | Yes | Yes | Yes | Yes |
| Quantitative Descriptive |  |  |  |  |  |  |  |  |
|  |  | S1. Are there clear research questions or aims? | S2. Do the collected data allow to address the research questions? | Is the sampling strategy relevant to address the research question? | Is the sample representative of the target population? | Are the measurements appropriate? | Is the risk of nonresponse bias low? | Is the statistical analysis appropriate to answer the research question? |
| (Supramaniam, Grindley, and Pulver 2006) | Cancer mortality in Aboriginal people in New South Wales, Australia, 1994-2002 | Yes | Yes | Yes | Yes | Yes | Yes | Yes |
| (Read and HaYes 2019) | Do Indigenous patients in Australia’s Northern Territory present with more advanced colorectal cancer? A cohort study based on registry data | Yes | Yes | Yes | Yes | Yes | Yes | Yes |
| (Valery et al. 2020) | Patterns of primary health care service use of Indigenous Australians diagnosed with cancer | Yes | Yes | Yes | Yes | Yes | Yes | Yes |
| (Cramb et al. 2012) | The first year counts: cancer survival among Indigenous and non-Indigenous Queenslanders, 1997–2006 | Yes | Yes | Yes | Yes | Yes | Yes | Yes |
| (Parikh et al. 2021) | The utilization of allied and community health services by cancer patients living in regional and remote geographical areas in Australia | Yes | Yes | Yes | Yes | Yes | Yes | Yes |
| (Bernardes et al. 2021) | End-of-life care for Aboriginal and Torres Strait Islander people with cancer: an exploratory study of service utilisation and unmet supportive care needs | Yes | Yes | Yes | Yes | Yes | Yes | Yes |
| (Bernardes et al. 2012) | Health service utilization by indigenous cancer patients in Queensland: a descriptive study | Yes | Yes | Yes | Yes | Yes | Yes | Yes |
| Qualitative |  |  |  |  |  |  |  |  |
|  |  | S1. Are there clear research questions or aims? | S2. Do the collected data allow to address the research questions? | Is the qualitative approach appropriate to answer the research question? | Are the qualitative data collection methods adequate to address the research question? | Are the findings adequately derived from the data? | Is the interpretation of results sufficiently substantiated by data? | Is there coherence between qualitative data sources, collection, analysis and interpretation? |
| (Newman et al. 2017) | ‘‘I Had a Little Bit of a Bloke MeltdownI But the Next Day, I Was Up’’: Understanding Cancer Experiences Among Aboriginal Men | Yes | Yes | Yes | Yes | Yes | Yes | Yes |
| (Shahid et al. 2011) | ‘Nowhere to room . . . nobody told them’: logistical and cultural impediments to Aboriginal peoples’ participation in cancer treatment | Yes | Yes | Yes | Yes | Yes | Yes | Yes |
| (Meiklejohn et al. 2020) | “I’m a Survivor”: Aboriginal and Torres Strait Islander Cancer Survivors’ Perspectives of Cancer Survivorship | Yes | Yes | Yes | Yes | Yes | Yes | Yes |
| (de Witt et al. 2018) | “It’s Just Presence,” the Contributions of Aboriginal and Torres Strait Islander Health Professionals in Cancer Care in Queensland | Yes | Yes | Yes | Yes | Yes | Yes | Yes |
| (Taylor et al. 2021) | “The support has been brilliant”: experiences of Aboriginal and Torres Strait Islander patients attending two high performing cancer services | Yes | Yes | Yes | Yes | Yes | Yes | Yes |
| (Bell et al. 2021) | “We Have to Be Strong Ourselves”: Exploring the Support Needs of Informal Carers of Aboriginal and Torres Strait Islander People with Cancer | Yes | Yes | Yes | Yes | Yes | Yes | Yes |
| (Cuesta-Briand et al. 2015) | Addressing unresolved tensions to build effective partnerships: lessons from an Aboriginal cancer support network | Yes | Yes | Yes | Yes | Yes | Yes | Yes |
| (Lyford et al. 2018) | An Exploration of Underrepresentation of Aboriginal Cancer Patients Attending a Regional Radiotherapy Service in Western Australia | Yes | Yes | Yes | Yes | Yes | Yes | Yes |
| (Shahid, Finn, Thompson, et al. 2009) | Barriers to participation of Aboriginal people in cancer care: communication in the hospital setting | Yes | Yes | Yes | Yes | Yes | Yes | Yes |
| (Olver et al. 2022) | Communicating cancer and its treatment to Australian Aboriginal and Torres Strait Islander patients with cancer: a qualitative study | Yes | Yes | Yes | Yes | Yes | Yes | Yes |
| (de Witt et al. 2020) | Communication, Collaboration and Care Coordination: The Three-Point Guide to Cancer Care Provision for Aboriginal and Torres Strait Islander Australians | Yes | Yes | Yes | Yes | Yes | Yes | Yes |
| (Marcusson-Rababi et al. 2019) | Does gynaecological cancer care meet the needs of Indigenous Australian women? Qualitative interviews with patients and care providers | Yes | Yes | Yes | Yes | Yes | Yes | Yes |
| (Tam et al. 2018) | Exploring Positive Survivorship Experiences of Indigenous Australian Cancer Patients | Yes | Yes | Yes | Yes | Yes | Yes | Yes |
| (Gonzalez et al. 2020) | Exploring the barriers preventing Indigenous Australians from accessing cancer genetic counselling | Yes | Yes | Yes | Yes | Yes | Yes | Yes |
| (Dembinsky 2014) | Exploring Yamatji perceptions and use of palliative care: an ethnographic study | Yes | Yes | Yes | Yes | Yes | Yes | Yes |
| (Shahid et al. 2016) | Factors contributing to delayed diagnosis of cancer among Aboriginal people in Australia: a qualitative study | Yes | Yes | Yes | Yes | Yes | Yes | Yes |
| (Meiklejohn et al. 2017) | Follow-up cancer care: perspectives of Aboriginal and Torres Strait Islander cancer survivors | Yes | Yes | Yes | Yes | Yes | Yes | Yes |
| (Meiklejohn et al. 2016) | Health professional’s perspectives the barriers and enablers cancer care Indigenous Australians | Yes | Yes | Yes | Yes | Yes | Yes | Yes |
| (Ivers et al. 2019) | Home to health care to hospital: Evaluation of a cancer care team based in Australian Aboriginal primary care | Yes | Yes | Yes | Yes | Yes | Yes | Yes |
| (Shahid et al. 2013) | Identifying barriers and improving communication between cancer service providers and Aboriginal patients and their families: the perspective of service providers | Yes | Yes | Yes | Yes | Yes | Yes | Yes |
| (Finn et al. 2008) | Improving Indigenous access to cancer screening and treatment services: descriptive findings and a preliminary report on the Midwest Indigenous Women’s Cancer Support Group | Yes | Yes | Yes | Yes | Yes | Yes | Yes |
| (Willis et al. 2011) | Indigenous women’s expectations of clinical care during treatment for a gynaecological cancer: rural and remote differences in expectations | Yes | Yes | Yes | Yes | Yes | Yes | Yes |
| (McMichael et al. 2000) | Indigenous women’s perceptions of breast cancer diagnosis and treatment in Queensland | Yes | Yes | Yes | Yes | Yes | Yes | Yes |
| (McGrath et al. 2006) | Insights on Aboriginal peoples’ views of cancer in Australia | Yes | Yes | Yes | Yes | Yes | Yes | Yes |
| (McGrath and Rawson 2013) | Key factors impacting on diagnosis and treatment for vulvar cancer for Indigenous women: findings from Australia | Yes | Yes | Yes | Yes | Yes | Yes | Yes |
| (Thompson et al. 2011) | Not just bricks and mortar: planning hospital cancer services for Aboriginal people | Yes | Yes | Yes | Yes | Yes | Yes | Yes |
| (Thompson et al. 2019) | Passing on wisdom: exploring the end-of-life wishes of Aboriginal people from the Midwest of Western Australia | Yes | Yes | Yes | Yes | Yes | Yes | Yes |
| (Prior 2009) | The meaning of cancer for Australian Aboriginal women; changing the focus of cancer nursing | Yes | Yes | Yes | Yes | Yes | Yes | Yes |
| (Ristevski et al. 2020) | Understanding Aboriginal Peoples’ Cultural and Family Connections Can Help Inform the Development of Culturally Appropriate Cancer Survivorship Models of Care | Yes | Yes | Yes | Yes | Yes | Yes | Yes |
| (Shahid, Finn, Bessarab, et al. 2009) | Understanding, beliefs and perspectives of Aboriginal people in Western Australia about cancer and its impact on access to cancer services | Yes | Yes | Yes | Yes | Yes | Yes | Yes |
| (Meiklejohn et al. 2019) | We just don't talk about it': Aboriginal and Torres Strait Islander peoples' perceptions of cancer in regional Queensland | Yes | Yes | Yes | Yes | Yes | Yes | Yes |
| Mixed Methods |  |  |  |  |  |  |  |  |
|  |  | S1. Are there clear research questions or aims? | S2. Do the collected data allow to address the research questions? | Is there an adequate rationale for using a mixed methods design to address the research question? | Are the different components of the study effectively integrated to answer the research question? | Are the outputs of the integration of qualitative and quantitative components adequately interpreted? | Are divergences and inconsistencies between quantitative and qualitative results adequately addressed? | Do the different components of the study adhere to the quality criteria of each tradition of the methods involved? |
| (Page et al. 2016) | A survey of lung cancer in rural and remote Aboriginal and Torres Strait Islander communities in Queensland: health views that impact on early diagnosis and treatment | Yes | Yes | Yes | Yes | Yes | No | Not sure |
| (Anderson et al. 2021) | Accessibility of cancer treatment services for Indigenous Australians in the Northern Territory: perspectives of patients and care providers | Yes | Yes | Yes | Yes | No | No | Not sure |

**Full References**

Anderson, Kate, Abbey Diaz, Darshit Rajeshkumar Parikh, and Gail Garvey. 2021. 'Accessibility of cancer treatment services for Indigenous Australians in the Northern Territory: Perspectives of patients and care providers', *BMC Health Services Research*, 21: 1-13.

Baade, Peter D., Paramita Dasgupta, Paul W. Dickman, Susanna Cramb, John D. Williamson, John R. Condon, and Gail Garvey. 2016. 'Quantifying the changes in survival inequality for Indigenous people diagnosed with cancer in Queensland, Australia', *Cancer Epidemiology*, 43: 1-8.

Banham, David, David Roder, and Alex Brown. 2018. 'Comorbidities contribute to the risk of cancer death among Aboriginal and non-Aboriginal South Australians: Analysis of a matched cohort study', *Cancer Epidemiology*, 52: 75-82.

Banham, David, David Roder, Marion Eckert, Natasha J. Howard, Karla Canuto, Alex Brown, D. A. D. Aboriginal Community Reference Group Can, and D. A. D. Investigators other Can. 2019. 'Cancer treatment and the risk of cancer death among Aboriginal and non-Aboriginal South Australians: Analysis of a matched cohort study', *BMC Health Services Research*, 19: 771.

Basnayake, Thilini L., Patricia C. Valery, Philip Carson, and Paolo B. De Ieso. 2021. 'Treatment and outcomes for Indigenous and non-Indigenous lung cancer patients in the Top End of the Northern Territory', *Internal Medicine Journal*, 51: 1081-91.

Bell, Lorraine, Kate Anderson, Afaf Girgis, Samar Aoun, Joan Cunningham, Claire E. Wakefield, Shahid Shaouli, Smith Allan Ben, Abbey Diaz, Daniel Lindsay, Masa Adam, and Gail Garvey. 2021. '“We Have to Be Strong Ourselves”: Exploring the support needs of informal carers of Aboriginal and Torres Strait Islander people with cancer', *International Journal of Environmental Research and Public Health*, 18: 7281.

Bernardes, C. M., D. Langbecker, V. Beesley, G. Garvey, and P. C. Valery. 2019. 'Does social support reduce distress and worry among Aboriginal and Torres Strait Islander people with cancer?', *Cancer Reports*, 2.

Bernardes, Christina M., Vanessa Beesley, Shahid Shaouli, Linda Medlin, Gail Garvey, and Patricia C. Valery. 2021. 'End-of-life care for Aboriginal and Torres Strait Islander people with cancer: An exploratory study of service utilisation and unmet supportive care needs', *Supportive care in cancer*, 29: 2073-82.

Bernardes, Christina M., Abbey Diaz, Patricia C. Valery, Sabe Sabesan, Siddhartha Baxi, Samar Aoun, Sandra C. Thompson, Mari Lashbrook, and Gail Garvey. 2019. 'Unmet supportive care needs among Indigenous cancer patients across Australia', *Rural & Remote Health*, 19: 1-11.

Bernardes, Christina M., Lisa J. Whop, Gail Garvey, and Patricia C. Valery. 2012. 'Health service utilization by Indigenous cancer patients in Queensland: A descriptive study', *International Journal for Equity in Health*, 11: 57-65.

Carruthers, Scott, Mary Pennefather, Linda Ward, Kar Giam, and Michael Penniment. 2019. 'Measuring (and narrowing) the gap: The experience with attendance of Indigenous cancer patients for Radiation Therapy in the Northern Territory', *Journal of Medical Imaging and Radiation Oncology*, 63: 510-16.

Condon, J. R., B. K. Armstrong, T. Barnes, and Y. J. Zhao. 2005. 'Cancer incidence and survival for Indigenous Australians in the northern territory', *Australian and New Zealand Journal of Public Health*, 29: 123-28.

Condon, John R., Tony Barnes, Bruce K. Armstrong, Sid Selva-Nayagam, and J. Mark Elwood. 2005. 'Stage at diagnosis and cancer survival for Indigenous Australians in the Northern Territory', *Medical Journal of Australia*, 182: 277-80.

Coory, Michael D., Adele C. Green, Janelle Stirling, and Patricia C. Valery. 2008. 'Survival of Indigenous and non-Indigenous Queenslanders after a diagnosis of lung cancer: A matched cohort study', *Medical Journal of Australia*, 188: 562-6.

Cottrell, Jennifer, Jackie Street, Alwin Chong, and David Roder. 2007. 'Comparing cancer profiles and survival of aboriginal and non-aboriginal patients in South Australia: where are the opportunities for improving Aboriginal health?', *Asian Pacific journal of cancer prevention : APJCP*, 8: 495-501.

Cramb, S. M., G. Garvey, P. C. Valery, J. D. Williamson, P. D. Baade, Susanna M. Cramb, Gail Garvey, Patricia C. Valery, John D. Williamson, and Peter D. Baade. 2012. 'The first year counts: cancer survival among Indigenous and non-Indigenous Queenslanders, 1997-2006', *Medical Journal of Australia*, 196: 270-74.

Cuesta-Briand, Beatriz, Dawn Bessarab, Shahid Shaouli, and Sandra C. Thompson. 2015. 'Addressing unresolved tensions to build effective partnerships: Lessons from an Aboriginal cancer support network', *International Journal for Equity in Health*, 14.

de Witt, Audra, Frances C. Cunningham, Ross Bailie, Nikki Percival, Jon Adams, and Patricia C. Valery. 2018. '"It's Just Presence," the contributions of Aboriginal and Torres Strait Islander health professionals in cancer care in Queensland', *Frontiers in Public Health*, 6: 344.

de Witt, Audra, Veronica Matthews, Ross Bailie, Gail Garvey, Patricia C. Valery, Jon Adams, Jennifer H. Martin, and Frances C. Cunningham. 2020. 'Communication, collaboration and care coordination: The three-point guide to cancer care provision for Aboriginal and Torres Strait Islander Australians', *International Journal of Integrated Care (IJIC)*, 20: 1-16.

Dembinsky, Melanie. 2014. 'Exploring Yamatji perceptions and use of palliative care: An ethnographic study', *International Journal of Palliative Nursing*, 20: 387-93.

Diaz, A., P. D. Baade, P. C. Valery, L. J. Whop, S. P. Moore, J. Cunningham, G. Garvey, J. M. L. Brotherton, D. L. O’Connell, K. Canfell, D. Sarfati, D. Roder, E. Buckley, and J. R. Condon. 2018. 'Comorbidity and cervical cancer survival of Indigenous and non-Indigenous Australian women: A semi-national registry-based cohort study (2003-2012)', *PLoS ONE [Electronic Resource]*, 13.

Diaz, A., C. M. Bernardes, G. Garvey, and P. C. Valery. 2016. 'Supportive care needs among Indigenous cancer patients in Queensland, Australia: Less comorbidity is associated with greater practical and cultural unmet need', *European Journal of Cancer Care*, 25: 242-53.

Diaz, Abbey, Suzanne P. Moore, Jennifer H. Martin, Adele C. Green, Gail Garvey, and Patricia C. Valery. 2015. 'Factors associated with cancer-specific and overall survival among Indigenous and non-Indigenous gynecologic cancer patients in Queensland, Australia: A matched cohort study', *International Journal of Gynecological Cancer* 25: 542-47.

Elder-Robinson, E., A. Diaz, K. Howard, D. R. Parikh, G. Kar, and G. Garvey. 2022. 'Quality of life in the first year of cancer diagnosis among Aboriginal and non-Aboriginal people living in regional and remote areas of Australia', *International Journal of Environmental Research and Public Health*, 19: 10.

Finn, L. D., A. Pepper, P. Gregory, and S. C. Thompson. 2008. 'Improving Indigenous access to cancer screening and treatment services: Descriptive findings and a preliminary report on the Midwest Indigenous Women's Cancer Support Group', *Australasian Medical Journal*, 1.

Fitzadam, S., E. M. Lin, N. Creighton, and D. C. Currow. 2021. 'Lung, breast and bowel cancer treatment for Aboriginal people in New South Wales: A population-based cohort study', *Internal Medicine Journal*, 51: 879-90.

Frydrych, A. M., L. M. Slack-Smith, R. Parsons, and T. Threlfall. 2014. 'Oral cavity squamous cell carcinoma: Characteristics and survival in Aboriginal and non-Aboriginal Western Australians', *The open dentistry journal*, 8: 168-74.

Garvey, G., J. Cunningham, V. Yf He, M. Janda, P. Baade, S. Sabesan, J. H. Martin, M. Fay, J. Adams, S. Kondalsamy-Chennakesavan, and P. C. Valery. 2016. 'Health-related quality of life among Indigenous Australians diagnosed with cancer', *Quality of Life Research*, 25: 1999-2008.

Gibberd, A., R. Supramaniam, A. Dillon, B. K. Armstrong, and D. L. O’Connell. 2015. 'Are Aboriginal people more likely to be diagnosed with more advanced cancer?', *Medical Journal of Australia*, 202: 195-200.

Gibberd, Alison, Rajah Supramaniam, Anthony Dillon, Bruce K. Armstrong, and Dianne L. O'Connell. 2016. 'Lung cancer treatment and mortality for Aboriginal people in New South Wales, Australia: Results from a population-based record linkage study and medical record audit', *BMC Cancer*, 16: 1-11.

Gonzalez, Tina, Rebecca Harris, Rachel Williams, Rose Wadwell, Kristine Barlow‐Stewart, Jane Fleming, and Melissa Buckman. 2020. 'Exploring the barriers preventing Indigenous Australians from accessing cancer genetic counseling', *Journal of Genetic Counseling*, 29: 542-52.

Hall, Sonja E, Caroline E. Bulsara, Max K. Bulsara, Timothy G. Leahy, Margaret R. Culbong, Delia Hendrie, and Arcy J. Holman. 2004. 'Treatment patterns for cancer in Western Australia: does being Indigenous make a difference?', *Medical Journal of Australia*, 181: 191-4.

Ho-Huynh, Albert Hoang Nguyen, Timothy J. Elston, Ronny K. Gunnarsson, and Alan De Costa. 2022. 'Achieving high breast cancer survival for women in rural and remote areas', *Asian Pacific Journal of Cancer Prevention*, 23: 101-07.

Ivers, Rowena, Brad Jackson, Trish Levett, Kyla Wallace, and Scott Winch. 2019. 'Home to health care to hospital: Evaluation of a cancer care team based in Australian Aboriginal primary care', *Australian Journal of Rural Health*, 27: 88-92.

Jessop, S., S. Ruhayel, R. Sutton, D. R. Youlden, G. Pearson, C. Lu, S. Milne, M. J. Henderson, J. F. Aitken, R. S. Kotecha, and T. Revesz. 2021. 'Are outcomes for childhood leukaemia in Australia influenced by geographical remoteness and Indigenous race?', *Pediatric Blood & Cancer*, 68: 8.

Laurvick, C. L., J. B. Semmens, D. J. Holman, and Y. C. Leung. 2003. 'Ovarian cancer in Western Australia (1982-98): Incidence, mortality and survival', *Australian and New Zealand Journal of Public Health*, 27: 588-95.

Le, Hien, Michael Penniment, Scott Carruthers, Daniel Roos, Thomas Sullivan, and Siddhartha Baxi. 2013. 'Radiation treatment compliance in the Indigenous population: The pilot Northern Territory experience and future directions', *Journal of Medical Imaging & Radiation Oncology*, 57: 218-21.

Luke, C., E. Tracey, A. Stapleton, and D. Roder. 2010. 'Exploring contrary trends in bladder cancer incidence, mortality and survival: Implications for research and cancer control', *Internal Medicine Journal*, 40: 357-62.

Lyford, Marilyn, Margaret M. Haigh, Siddhartha Baxi, Shelley Cheetham, Shahid Shaouli, and Sandra C. Thompson. 2018. 'An exploration of underrepresentation of Aboriginal cancer patients attending a regional radiotherapy service in Western Australia', *International Journal of Environmental Research and Public Health*, 15: 337.

Marcusson-Rababi, Beverley, Kate Anderson, Lisa J. Whop, Tamara Butler, Nicole Whitson, and Gail Garvey. 2019. 'Does gynaecological cancer care meet the needs of Indigenous Australian women? Qualitative interviews with patients and care providers', *BMC Health Services Research*, 19: N.PAG-N.PAG.

Martin, Jennifer H., M. D. Coory, P. C. Valery, and A. C. Green. 2009. 'Association of diabetes with survival among cohorts of Indigenous and non-Indigenous Australians with cancer', *Cancer Causes & Control*, 20: 355-60.

McGrath, P., H. Holewa, K. Ogilvie, R. Rayner, and M. A. Patton. 2006. 'Insights on Aboriginal peoples' views of cancer in Australia', *Contemporary Nurse: A Journal for the Australian Nursing Profession*, 22: 240-54.

McGrath, Pam, and Nicole Rawson. 2013. 'Key factors impacting on diagnosis and treatment for vulvar cancer for Indigenous women: Findings from Australia', *Supportive care in cancer*, 21: 2769-75.

McMichael, Celia, Maureen Kirk, Lenore Manderson, Elizabeth Hoban, and Helen Potts. 2000. 'Indigenous women's perceptions of breast cancer diagnosis and treatment in Queensland', *Australian and New Zealand Journal of Public Health*, 24: 515-19.

Meiklejohn, J. A., J. Adams, P. C. Valery, E. T. Walpole, J. H. Martin, H. M. Williams, and G. Garvey. 2016. 'Health professional's perspectives of the barriers and enablers to cancer care for Indigenous Australians', *European Journal of Cancer Care*, 25: 254-61.

Meiklejohn, J. A., G. Garvey, R. Bailie, E. Walpole, J. Adams, D. Williamson, J. Martin, C. M. Bernardes, B. Arley, B. Marcusson, and P. C. Valery. 2017. 'Follow-up cancer care: perspectives of Aboriginal and Torres Strait Islander cancer survivors', *Supportive care in cancer*, 25: 1597-605.

Meiklejohn, Judith A. , Ross Bailie, Jon Adams, Gail Garvey, Christina M Bernardes, Daniel Williamson, Beverly Marcusson, Brian B. Arley, Jennifer H. Martin, Euan T. Walpole, and Patricia C. Valery. 2020. '"I'm a Survivor": Aboriginal and Torres Strait Islander cancer survivors' perspectives of cancer survivorship', *Cancer Nursing*, 43: 105.

Meiklejohn, Judith A., Brian D. Arley, Gregory Pratt, Patricia C. Valery, and Christina M. Bernardes. 2019. ''We just don't talk about it': Aboriginal and Torres Strait Islander peoples' perceptions of cancer in regional Queensland', *Rural & Remote Health*, 19: 1-9.

Moore, Suzanne P., Adèle C. Green, Freddie Bray, Gail Garvey, Michael Coory, Jennifer Martin, and Patricia C. Valery. 2014. 'Survival disparities in Australia: an analysis of patterns of care and comorbidities among indigenous and non-indigenous cancer patients', *BMC Cancer*, 14: 517-17.

Moore, Suzanne P., Adèle C. Green, Gail Garvey, Michael D. Coory, and Patricia C. Valery. 2011. 'A study of head and neck cancer treatment and survival among Indigenous and non-Indigenous people in Queensland, Australia, 1998 to 2004', *BMC Cancer*, 11: 460-60.

Moore, Suzanne P., Isabelle Soerjomataram, Adèle C. Green, Gail Garvey, Jennifer Martin, and Patricia C. Valery. 2016. 'Breast cancer diagnosis, patterns of care and burden of disease in Queensland, Australia (1998-2004): does being Indigenous make a difference?', *International Journal of Public Health*, 61: 435-42.

Newman, Christy E., Rebecca Gray, Loren Brener, L. Clair Jackson, Anthony Dillon, Veronica Saunders, Priscilla Johnson, and Carla Treloar. 2017. '''I had a little bit of a bloke meltdown...But the next day, I was up'': Understanding cancer experiences among Aboriginal men', *Cancer Nursing*, 40: E1-E8.

Olver, Ian, Kate M. Gunn, Alwin Chong, Vikki Knott, Kristiaan Spronk, Cominos Nayia, and Joan Cunningham. 2022. 'Communicating cancer and its treatment to Australian Aboriginal and Torres Strait Islander patients with cancer: A qualitative study', *Supportive care in cancer*, 30: 431-38.

Page, B. J., R. V. Bowman, I. A. Yang, and K. M. Fong. 2016. 'A survey of lung cancer in rural and remote Aboriginal and Torres Strait Islander communities in Queensland: Health views that impact on early diagnosis and treatment', *Internal Medicine Journal*, 46: 171-76.

Parikh, Darshit Rajeshkumar, Abbey Diaz, Christina Bernardes, Paolo B. De Ieso, Thanuja Thachil, Giam Kar, Matthew Stevens, and Gail Garvey. 2021. 'The utilization of allied and community health services by cancer patients living in regional and remote geographical areas in Australia', *Supportive care in cancer*, 29: 3209-17.

Parker, Christopher, Steven Y. C. Tong, Karen Dempsey, John Condon, Suresh K. Sharma, John W. C. Chen, William Sievert, and Joshua S. Davis. 2014. 'Hepatocellular carcinoma in Australia's Northern Territory: High incidence and poor outcome', *Medical Journal of Australia*, 201: 470-74.

Prior, D. 2009. 'The meaning of cancer for Australian Aboriginal women; changing the focus of cancer nursing', *European Journal of Oncology Nursing*, 13: 280-86.

Pule, Lettie, Elizabeth Buckley, Theophile Niyonsenga, David Banham, and David Roder. 2018. 'Developing a comorbidity index for comparing cancer outcomes in Aboriginal and non-Aboriginal Australians', *BMC Health Services Research*, 18: N.PAG-N.PAG.

Read, David J., and Ian HaYes. 2019. 'Do Indigenous patients in Australia's Northern Territory present with more advanced colorectal cancer? A cohort study based on registry data', *ANZ Journal of Surgery*, 89: 1296-301.

Ristevski, Eli, Sharyn Thompson, Sharon Kingaby, Claire Nightingale, and Mahesh Iddawela. 2020. 'Understanding Aboriginal peoples' cultural and family connections can help inform the development of culturally appropriate cancer survivorship models of care', *JCO Global Oncology*, 6: 124-32.

Roder, David, Fleur Webster, Helen Zorbas, and Sue Sinclair. 2012. 'Breast screening and breast cancer survival in Aboriginal and Torres Strait Islander women of Australia', *Asian Pacific journal of cancer prevention : APJCP*, 13: 147-55.

Rodger, Jennifer C., Rajah Supramaniam, Alison J. Gibberd, David P. Smith, Bruce K. Armstrong, Anthony Dillon, and Dianne L. O'Connell. 2015. 'Prostate cancer mortality outcomes and patterns of primary treatment for Aboriginal men in New South Wales, Australia', *BJU International*, 115: 16-23.

Rotte, L., J. Hansford, M. Kirby, M. Osborn, R. Suppiah, P. Ritchie, H. Tapp, M. Rice, and T. Revesz. 2013. 'Cancer in Australian Aboriginal children: Room for improvement', *Journal of Paediatrics & Child Health*, 49: 27-32.

Shahid, S., L. Finn, D. Bessarab, S. C. Thompson, Shaouli Shahid, Lizzie Finn, Dawn Bessarab, and Sandra C. Thompson. 2009. 'Understanding, beliefs and perspectives of Aboriginal people in Western Australia about cancer and its impact on access to cancer services', *BMC Health Services Research*, 9: 132-32.

Shahid, S., L. D. Finn, S. C. Thompson, Shaouli Shahid, Lizzie D. Finn, and Sandra C. Thompson. 2009. 'Barriers to participation of Aboriginal people in cancer care: Communication in the hospital setting', *Medical Journal of Australia*, 190: 574-79.

Shahid, S., T. H. K. Teng, D. Bessarab, S. Aoun, S. Baxi, and S. C. Thompson. 2016. 'Factors contributing to delayed diagnosis of cancer among Aboriginal people in Australia: A qualitative study', *BMJ Open*, 6: 11.

Shahid, Shaouli, Angela Durey, Dawn Bessarab, Samar M. Aoun, and Sandra C. Thompson. 2013. 'Identifying barriers and improving communication between cancer service providers and Aboriginal patients and their families: The perspective of service providers', *BMC Health Services Research*, 13: 460-60.

Shahid, Shaouli, Lizzie Finn, Dawn Bessarab, and Sandra Thompson. 2011. ''Nowhere to room . . . nobody told them': Logistical and cultural impediments to Aboriginal peoples' participation in cancer treatment', *Australian Health Review*, 35: 235-41.

Shaw, Ian M., and Timothy J. Elston. 2003. 'Retrospective, 5-year surgical audit comparing breast cancer in Indigenous and non-Indigenous women in Far North Queensland', *ANZ Journal of Surgery*, 73: 758-60.

Slape, D. R. , R. B. Saunderson, A. H. Tatian, D. F. Forstner, and V. J. Estall. 2019. 'Cutaneous malignancies in Indigenous Peoples of urban Sydney', *Journal of Medical Imaging and Radiation Oncology*, 63: 244-49.

Supramaniam, R., H. Grindley, and L. J. Pulver. 2006. 'Cancer mortality in Aboriginal people in New South Wales, Australia, 1994-2002', *Australian & New Zealand Journal of Public Health*, 30: 453-56.

Supramaniam, Rajah, Alison Gibberd, Anthony Dillon, David Eamon Goldsbury, and Dianne L. O'Connell. 2014. 'Increasing rates of surgical treatment and preventing comorbidities may increase breast cancer survival for Aboriginal women', *BMC Cancer*, 14: 163-63.

Tam, Laura, Gail Garvey, Judith Meiklejohn, Jennifer Martin, Jon Adams, Euan Walpole, Michael Fay, and Patricia Valery. 2018. 'Exploring positive survivorship experiences of Indigenous Australian cancer patients', *International Journal of Environmental Research and Public Health*, 15: 135.

Tan, J. Y. , Z. A. Otty, V. N. Vangaveti, P. Buttner, S. C. Varma, A. J. Joshi, J. Kelly, M. Collins, and S. S. Sabesan. 2016. 'A prospective comparison of times to presentation and treatment of regional and remote head and neck patients in North Queensland, Australia', *Internal Medicine Journal*, 46: 917-24.

Taylor, Emma V., Marilyn Lyford, Michele Holloway, Lorraine Parsons, Toni Mason, Sabe Sabesan, and Sandra C. Thompson. 2021. '"The support has been brilliant": experiences of Aboriginal and Torres Strait Islander patients attending two high performing cancer services', *BMC Health Services Research*, 21: 1-15.

Tervonen, Hanna E., Sanchia Aranda, David Roder, Richard Walton, Deborah Baker, Hui You, and David Currow. 2016. 'Differences in impact of Aboriginal and Torres Strait Islander status on cancer stage and survival by level of socio-economic disadvantage and remoteness of residence-A population-based cohort study in Australia', *Cancer Epidemiology*, 41: 132-38.

Tervonen, Hanna E., Richard Walton, Hui You, Deborah Baker, David Roder, David Currow, and Sanchia Aranda. 2017. 'After accounting for competing causes of death and more advanced stage, do Aboriginal and Torres Strait Islander peoples with cancer still have worse survival? A population-based cohort study in New South Wales', *BMC Cancer*, 17.

Thompson, S., M. Lyford, L. Papertalk, and M. Holloway. 2019. 'Passing on wisdom: Exploring the end-of-life wishes of Aborginal people from the Midwest of western Australia', *Rural and remote health*, 19: 9.

Thompson, Sandra C., Shaouli Shahid, Dawn Bessarab, Angela Durey, and Patricia M. Davidson. 2011. 'Not just bricks and mortar: Planning hospital cancer services for Aboriginal people', *BMC Research Notes*, 4: 62.

Tomita, Yoko, Christos S. Karapetis, David Roder, Carol Beeke, Christopher Hocking, Amitesh C. Roy, Amanda R. Townsend, Rob Padbury, Guy Maddern, and Timothy J. Price. 2016. 'Comparable survival outcome of metastatic colorectal cancer in Indigenous and non-Indigenous patients: Retrospective analysis of the South Australian metastatic colorectal cancer registry', *Australian Journal of Rural Health*, 24: 85-91.

Valery, P. C., M. Coory, J. Stirling, and A. C. Green. 2006. 'Cancer diagnosis, treatment, and survival in Indigenous and non-Indigenous Australians: A matched cohort study', *Lancet*, 367: 1842-48.

Valery, Patricia C., Christina M. Bernardes, Vanessa Beesley, Anna L. Hawkes, Peter Baade, and Gail Garvey. 2017. 'Unmet supportive care needs of Australian Aboriginal and Torres Strait Islanders with cancer: A prospective, longitudinal study', *Supportive care in cancer*, 25: 869-77.

Valery, Patricia C., Christina M. Bernardes, Audra de Witt, Jennifer Martin, Euan Walpole, Gail Garvey, Daniel Williamson, Judith Meiklejohn, Gunter Hartel, Isanka U. Ratnasekera, and Ross Bailie. 2020. 'Patterns of primary health care service use of Indigenous Australians diagnosed with cancer', *Supportive care in cancer*, 28: 317-27.

Valery, Patricia C., Danny R. Youlden, Peter D. Baade, Leisa J. Ward, Adele C. Green, and Joanne F. Aitken. 2013a. 'Cancer incidence and mortality in Indigenous Australian children, 1997-2008', *Pediatric Blood & Cancer*, 60: 156-58.

———. 2013b. 'Cancer survival in Indigenous and non-Indigenous Australian children: What is the difference?', *Cancer Causes & Control*, 24: 2099-106.

Weir, K., R. Supramaniam, A. Gibberd, A. Dillon, B. K. Armstrong, and D. L. O’Connell. 2016. 'Comparing colorectal cancer treatment and survival for Aboriginal and non-Aboriginal people in New South Wales ', *Medical Journal of Australia*, 204: 156.e1-56.e8.

Whop, L. J., P. D. Baade, J. M. L. Brotherton, K. Canfell, J. Cunningham, D. Gertig, K. Lokuge, G. Garvey, S. P. Moore, A. Diaz, D. L. O’Connell, P. Valery, D. M. Roder, and J. R. Condon. 2017. 'Time to clinical investigation for Indigenous and non-Indigenous Queensland women after a high grade abnormal pap smear, 2000-2009', *Medical Journal of Australia*, 206: 73-77.

Willis, Eileen, Judith Dwyer, Kei Owada, Leah Couzner, Debra King, and Jo Wainer. 2011. 'Indigenous women's expectations of clinical care during treatment for a gynaecological cancer: Rural and remote differences in expectations', *Australian Health Review*, 35: 99-103.

Woods, J. A., C. E. Johnson, H. T. Ngo, J. M. Katzenellenbogen, K. Murray, and S. C. Thompson. 2018. 'Delay in commencement of palliative care service episodes provided to Indigenous and non-Indigenous patients: Cross-sectional analysis of an Australian multi-jurisdictional dataset', *BMC Palliative Care*, 17: 11.

Yang, Peng, and Peter Baade. 2021. 'Survival disparities among recently diagnosed Aboriginal and Torres Strait Islander cancer patients in Australia remain', *Cancer Causes & Control*, 32: 1315-20.

Youlden, Danny R., Peter D. Baade, Craig A. McBride, Jason D. Pole, Andrew S. Moore, Patricia C. Valery, Angela Young, and Joanne F. Aitken. 2022. 'Changes in cancer incidence and survival among Aboriginal and Torres Strait Islander children in Australia, 1997–2016', *Pediatric Blood & Cancer*, 69: e29492.
